# Crystallographic characterization of a marine invertebrate ferritin from the sea cucumber *Apostichopus japonicus*

**DOI:** 10.1002/2211-5463.13375

**Published:** 2022-02-07

**Authors:** Yan Wu, Tinghong Ming, Chunheng Huo, Xiaoting Qiu, Chang Su, Chenyang Lu, Jun Zhou, Ye Li, Xiurong Su

**Affiliations:** ^1^ 47862 State Key Laboratory for Managing Biotic and Chemical Threats to the Quality and Safety of Agro‐products Ningbo University China; ^2^ 47862 College of Food and Pharmaceutical Sciences Ningbo University China; ^3^ 47862 School of Marine Science Ningbo University China; ^4^ Zhejiang Collaborative Innovation Center for High Value Utilization of Byproducts from Ethylene Project Ningbo Polytechnic College China

**Keywords:** 3‐fold channel, *Apostichopus japonicus*, ferritin, ferroxidase center, metal transport pathway

## Abstract

Ferritin is considered to be an ubiquitous and conserved iron‐binding protein that plays a crucial role in iron storage, detoxification, and immune response. Although ferritin is of critical importance for almost all kingdoms of life, there is a lack of knowledge about its role in the marine invertebrate sea cucumber (*Apostichopus japonicus*). In this study, we characterized the first crystal structure of *A. japonicus* ferritin (AjFER) at 2.75 Å resolution. The structure of AjFER shows a 4‐3‐2 symmetry cage‐like hollow shell composed of 24 subunits, mostly similar to the structural characteristics of other known ferritin species, including the conserved ferroxidase center and 3‐fold channel. The 3‐fold channel consisting of three 3‐fold negative amino acid rings suggests a potential pathway in which metal ions can be first captured by Asp120 from the outside environment, attracted by His116 and Cys128 when entering the channel, and then transferred by Glu138 from the 3‐fold channel to the ferroxidase site. Overall, the presented crystal structure of AjFER may provide insights into the potential mechanism of the metal transport pathway for related marine invertebrate ferritins.

## Graphical abstract

In this study, we characterize the first crystal structure of *Apostichopus japonicus* ferritin (AjFER). In AjFER, the 3‐fold channel consisting of three 3‐fold negative amino acid rings suggests a potential pathway in which metal ions can be first captured by Asp120 from the outside environment, attracted by His116 and Cys128 when entering the channel, and then transferred by Glu138 from the 3‐fold channel to the ferroxidase site.

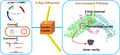

**Abbreviations**
AjFER: *Apostichopus japonicus* ferritin
CD: circular dichroism
ChF: *Chaetopterus* sp. ferritin
DLS: dynamic light scattering
H subunit: heavy subunit
HuHF: *Homo sapiens* ferritin H‐chain
HuLF: *Homo sapiens* ferritin L‐chain
ICP‐MS: inductively coupled plasma‐mass spectrometry
IPTG: isopropyl‐1‐thio‐β‐d‐galactoside
L subunit: light subunit
M subunit: middle subunit
MjFer: *Marsupenaeus japonicus* ferritin
SSRF: Shanghai Synchrotron Radiation Facility

Iron is an essential element for virtually all forms of cellular life, where it is crucial due to its critical involvement in many biological processes, for example, various redox reactions, cellular energy production, and others; importantly, this metal can serve as both a cofactor and catalyst in enzymatic reactions [1, 2]. Iron deficiency in organisms causes cessation of cell growth, leading to cell death, whereas iron overload is acutely toxic to cells owing to the increased generation of oxidative stress from labile iron [3]. Thus, for the cell growth of living organisms, it is crucial to maintain iron homeostasis. Ferritin, one of the major non‐heme iron storage and detoxification proteins, is widely found in most cell types of animals, plants, and microorganisms [4]. It is generally believed that ferritins are ancient proteins that can store iron in the form of a ferric oxide core and release iron again in a controlled fashion at the time of need [5, 6].

Ferritin is a multimeric protein with a nanocage structure that can accommodate up to approximately 4500–5000 ferric ions (Fe^3+^) inside the cage as a ferric oxyhydroxide cluster after ferrous ion (Fe^2+^) oxidation [4, 6]. In general, vertebrate ferritins are usually composed of heavy (H, ~ 21 kDa) and light (L, ~ 19 kDa) subunits having ~ 55% amino acid residue identity, which differ in the rates of iron uptake and mineralization [7, 8]. In particular, the H‐subunit typically comprises a di‐iron ferroxidase center, while the L‐subunit contains only a putative nucleation site for iron core formation [3, 7]. In mammals, the L‐ and H‐subunits can assemble into 24‐mer heteropolymers in various ratios depending on the organ or tissue, and in particular, these two subunits play cooperative roles in iron sequestration [9]. In addition, a third type of ferritin subunit, named the middle (M, ~ 20 kDa) subunit, has been identified in lower vertebrate (e.g., bullfrogs and fish) and invertebrate species [2, 10, 11]. The crystal structure of M‐type ferritin was described for the first time in amphibians, harboring both residues forming the ferroxidase center and negatively charged residues forming micelle nucleation site(s) [12]. Thus, M‐ferritin has functions of both efficient iron oxidation‐like mammalian H‐subunits and efficient iron mineralization‐like mammalian L‐subunits [11]. More recently, exploring the structural and functional features of ferritins in these ancient animals is of special interest.

Invertebrate ferritins can perform some unique functions that are found in vertebrate ferritins. Some studies have shown that ferritin‐like superfamily proteins are probably involved in innate immune defense in marine invertebrates [13, 14, 15]. Research has shown that ferritin of the starfish *Asterias forbesi* is considered to be an acute‐phase protein in iron sequestration, playing a crucial role in the invertebrate immune response [13]. It was found that the marine tubeworm *Chaetopterus* sp. ferritin (ChF) was associated with the production of bioluminescence in the secreted mucus [16]. Additionally, ChF has been proven to have a high rate of ferroxidase activity, up to eight times faster than that of *Homo sapiens* H‐chain ferritin (HuHF) [14]. Masuda et al. [15] purified *Marsupenaeus japonicus* ferritin (MjFer) and determined its crystal structure with a high resolution of 1.16 Å, demonstrating that MjFer exhibited a markedly higher binding capacity to Cd^2+^ and Hg^2+^ ions than HuHF [17]. Therefore, much more attention has been given to ferritin from marine invertebrate species.

The sea cucumber *Apostichopus japonicus* (Selenka) is an epibenthic species that is widely distributed along the coast of the northwestern Pacific region, and it has become an economically important aquaculture species among echinoderms [18]. Research has showed that *A. japonicus* ferritin (AjFER) can play crucial roles not only in innate immune defense by iron sequestration but also in the cellular iron homeostasis [19]. Moreover, Si et al. [20] demonstrated that AjFER exhibits a pronouncedly larger binding capacity to several kinds of heavy metal ions, probably owing to the presence of the key amino acid residues from the 3‐fold channels and ferroxidase center. Therefore, it is of biomedical importance to understand the structure and function of AjFER. In this study, we established a method for purification of AjFER and determined its crystal structure at a resolution of 2.75 Å. This method is expected to provide further insights into the underlying iron storage by ferritin in *A. japonicus* through a detailed structural picture.

## Materials and methods

### Sequence alignment and phylogenetic tree construction

Based on the amino acid sequences of AjFER [20], the theoretical isoelectric point (pI) and the predicted molecular weight (MW) were determined using the ExPASy server (http://web.expasy.org/peptide_mass/). The full‐length amino acid sequences of ferritin were obtained from NCBI GenBank (www.ncbi.nlm.nih.gov/genbank/). Multiple sequence alignments were performed using clustalw [21] and displayed using espript 3.0 [22]. The phylogenetic tree was constructed based on the neighbor‐joining method (1000 bootstrap replications) by mega 7.0 software [23].

### Protein preparation and iron content

Cloning of ferritin cDNA from *A. japonicus* was performed as described in detail in a previous report [24]. AjFER cDNA was 1222 bp in length with a 513 bp 5′‐UTR and 187 bp 3′‐UTR; it contained a 522‐bp complete open reading frame encoding a polypeptide with 173 amino acid residues [20]. The primers (forward: 5′‐CCGCTCGAGAATTAGGAGGAAGTCCAAGA‐3′ and reverse: 5′‐CGCCATATGTCGGACTCAGAAGTCAATCA‐3′) were designed based on the sequence of AjFER. The amplified PCR fragments were cloned into a pMD 18‐T vector (Takara, Dalian, China) and then digested using *Nde*I and *Xho*I (Takara) enzymes. They were subcloned into the *Nde*I/*Xho*I sites of the pET‐28a(+) expression vector (Novagen, Madison, WI, USA) with an N‐terminal His_6_‐SUMO tag to generate a recombinant AjFER plasmid. The plasmid was transformed into *Escherichia coli* Rosetta (DE3) cells (Novagen). The cells were cultured in Luria‐Bertani broth (LB) liquid medium supplemented with 30 μg·mL^−1^ kanamycin and 34 μg·mL^−1^ chloramphenicol until they were grown to an optical density at 600 nm (OD_600_) of 0.6 at 37 °C. Overexpression of the recombinant protein was induced for an additional 18 h with 0.5 mm isopropyl β‐d‐1‐thiogalactopyranoside (IPTG) at 18 °C.

The cells were harvested by centrifugation at 6680 *g* for 15 min at 4 °C, resuspended in lysis buffer [25 mm Tris–HCl, pH 8.0, 150 mm NaCl, 0.5% (v/v) Triton X‐100], and then disintegrated by sonication in a cooling ice bath. After centrifugation at 10 724 *g* for 20 min at 4 °C, the clear lysate was loaded onto a Ni–NTA affinity column (GE Healthcare, Fairfield, CT, USA) pre‐equilibrated with binding buffer (25 mm Tris–HCl, pH 8.0, 150 mm NaCl). Then, the protein was washed with wash buffer (25 mm Tris–HCl, pH 8.0, 150 mm NaCl, 70 mm imidazole) and eluted with elution buffer (25 mm Tris–HCl, pH 8.0, 150 mm NaCl, 500 mm imidazole). The protein was concentrated and loaded onto a Hi‐Load 16/600 Superdex 200 pg gel‐filtration chromatography column (GE Healthcare) equilibrated with binding buffer. After IPTG‐induced expression and multistage purification, AjFER containing an N‐terminal His_6_‐SUMO tag was obtained. The His_6_‐SUMO tag of AjFER was digested with SUMO protease overnight for processing at 4 °C using a 1 : 500 ratio of protease to protein and was removed using a Ni‐NTA affinity column (GE Healthcare). The purified proteins were concentrated using a stirred ultrafiltration cell with a 30 kDa molecular weight cutoff (MWCO) membrane filter (Millipore, Billerica, MA, USA). The purity of AjFER was analyzed by 12% SDS/PAGE. The protein concentration was determined using a bicinchoninic acid assay kit (Beyotime, Shanghai, China).

The AjFER sample (approx. 1.0 mg·mL^−1^) was prepared and digested using microwave digestion (MARS 5; CEM Corp., Matthews, NC, USA). The iron content of AjFER was measured by inductively coupled plasma‐mass spectrometry (ICP‐MS) using a Thermo X Series II ICP‐MS instrument (Thermo Fisher Scientific Inc., Waltham, MA, USA) as previously described [20].

### Dynamic light scattering analysis

Dynamic light scattering (DLS) data were collected on a Zetasizer Nano Zs instrument (Malvern Instruments Ltd., Malvern, Worcestershire, UK) in disposable polystyrene micro‐cuvettes (VWR) using 1500 μL of the freshly prepared 1 mg·mL^−1^ protein solution in binding buffer (25 mm Tris–HCl, pH 8.0, 150 mm NaCl) at 25 °C [25]. Three measurements were performed with the instrument optimizing the number of runs for each measurement. omnisize 2.0 software (Malvern Instruments Ltd., Malvern, Worcestershire, UK) was used to calculate the size distribution of protein samples.

### Circular dichroism spectroscopy analysis

The circular dichroism (CD) spectra of AjFER were measured from 260 to 190 nm using a Jasco J‐1700 CD spectrometer (Jasco, Tokyo, Japan) at room temperature in a 0.1 cm path length cuvette [20]. The nitrogen flow rate was 5 L·min^−1^, and binding buffer was used as the reference solution. The protein concentration was adjusted to 0.25 mg·mL^−1^ with binding buffer (25 mm Tris–HCl, pH 8.0, 150 mm NaCl). The CD data were plotted as molar ellipticity (deg·cm^2^·dmol^−1^) versus wavelength (λ). The proportions of α‐helices, β‐sheets, β‐turns, and random coils were analyzed using CD software.

### Crystallization, data collection, and structure determination

Crystals of AjFER were prepared using the sitting‐drop vapor‐diffusion method in crystallization plates at 18 °C by mixing 1 µL of 20 mg·mL^−1^ protein with 1 µL of a reservoir solution using Crystal Screen kits I and II (Hampton Research, Aliso Viejo, CA, USA). Clusters of cubical‐like crystals (dimensions ranging between 0.2 and 0.3 mm) with good diffraction quality were obtained from the optimized reservoir solution (0.2 m magnesium acetate tetrahydrate, 0.1 m sodium cacodylate trihydrate pH 6.5, 30% v/v(+/−)‐2‐methyl‐2,4‐pentanediol). The crystals of AjFER were separated and mounted in CryoLoops (Hampton Research) and quickly soaked in reservoir solution supplemented with 20% (v/v) glycerol as a cryoprotectant solution. The crystals were flash‐frozen in liquid nitrogen for data collection.

X‐ray diffraction data were collected at 100 K on the BL17U1 beamline at the Shanghai Synchrotron Radiation Facility (SSRF, Shanghai, China) using an EIGER X 16 M detector [26]. The X‐ray wavelength was set to 1.55 Å. Integration, scaling, and merging of the diffraction data were processed using the hkl‐2000 suite [27]. The AjFER structure was determined by molecular replacement using the online version of bables [28]. The structure was refined using the program refmac5 [29] as implemented in the CCP4 suite. Model building and structure adjustments were performed based on the sigma weighted (2*F*o − *F*c) and (*F*o − *F*c) electron density maps by using the program coot [15, 30]. Water molecules were located at the well‐defined positive electron density with a lower cutoff of 3σ in the (*F*o − *F*c) map. Metal ions were positioned into higher (*F*o − *F*c) residual densities and were based on shorter bond distances with neighboring water molecules or other protein ligands [31]. All crystallographic figures were generated with the program pymol, and surface electrostatic potentials were calculated using the Adaptive Poisson Boltzmann Solver (APBS) [32]. The statistics of data collection and structure refinement are summarized in Table 1. Superpositioning of AjFER with other structures was performed using the online Superpose server (http://superpose.wishartlab.com/) [33].

**Table 1:** Data collection and refinement statistics. Values in parentheses correspond to the highest resolution shell.

Crystal parameters	AjFER
Data collection	
Beamline	SSRF‐BL17U1
Wavelength (Å)	1.55
Space group	P12_1_1
Resolution range (Å)	50.0–2.75 (2.80–2.75)
Cell dimensions	
*a*, *b*, *c* (Å)	126.8, 178.8, 126.7
α, β, γ (°)	90, 90, 90
Number of all observations	494 454
Number of unique reflections	142 777
Completeness (%)	98.4 (94.7)
Mean *I*/σ(*I*)	9.7 (1.7)
*R*_merge_ (%)	12.3 (55.7)
Redundancy	3.5 (3.1)
CC_1/2_	0.8 (0.7)
Refinement	
*R*_work_/*R*_free_ (%)	19.0/26.5
R.m.s. deviations	
Bond lengths (Å)	0.008
Bond angles (°)	1.18
Ramachandran plot	
Favored regions (%)	97.0
Allowed regions (%)	2.8
Outlier (%)	0.2
PDB ID	7VHR

## Results

### Homology analysis and general characterization of AjFER

**Fig. 1:**
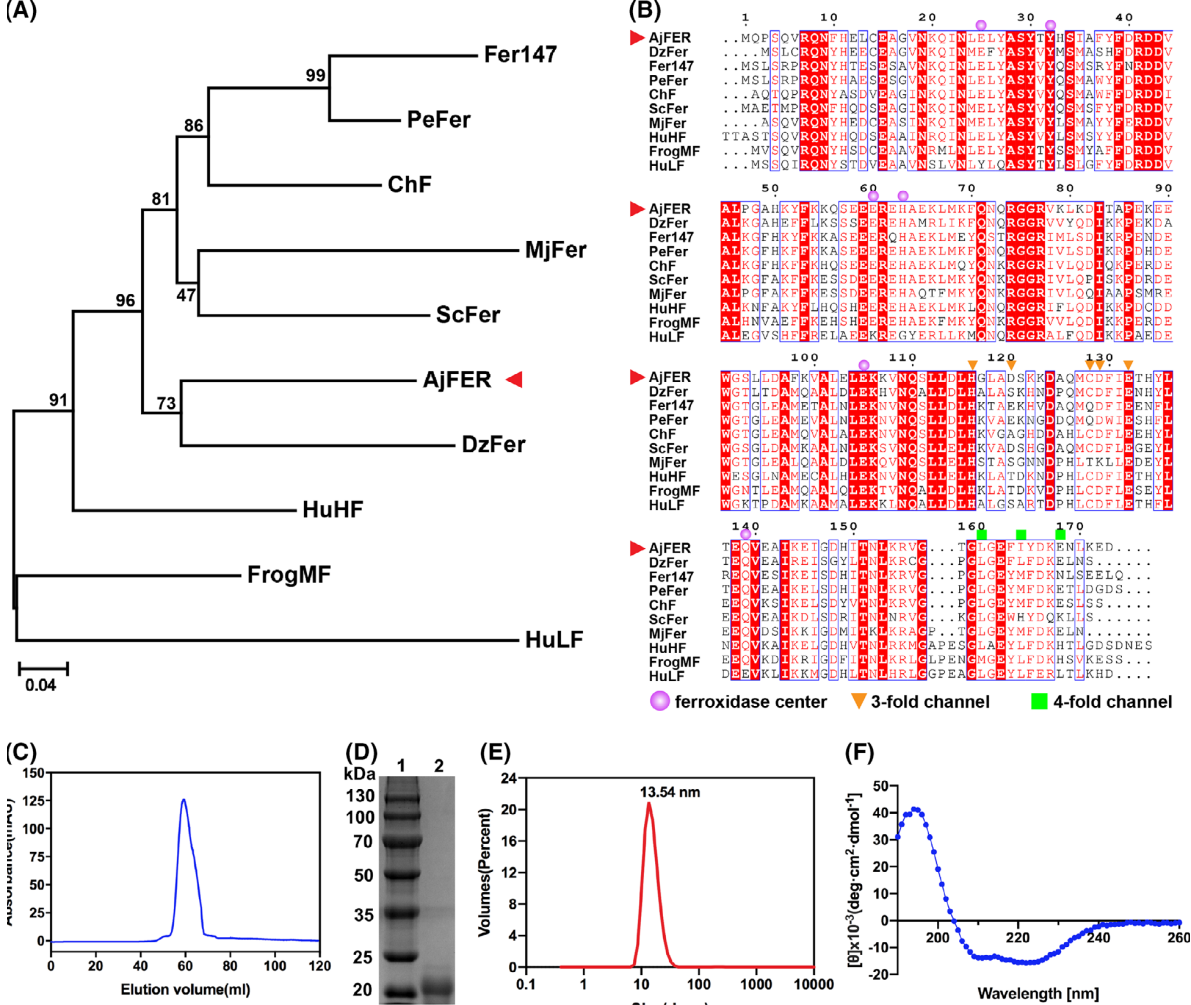
Homology analysis and characterization of AjFER. (A) Phylogenetic tree diagram. (B) Multiple sequence alignment of *Apostichopus japonicus* ferritin and nine other ferritin polypeptides. AjFER, *Apostichopus japonicus* ferritin; FrogMF, *Rana catesbeiana* M ferritin; HuHF, *Homo sapiens* H chain; HuLF, *Homo sapiens* L chain; ChF, *Chaetopterus* sp. ferritin; MjFer, *Marsupenaeus japonicus* ferritin; Fer147, novel *Phascolosoma esculenta* ferritin; PeFer, *Phascolosoma esculenta* ferritin; ScFer, *Sinonovacula constricta* ferritin; DzFer, *Dendrorhynchus zhejiangensis* ferritin. (C) Superdex 200 gel filtration chromatography profile of AjFER. The absorbance at 280 nm is shown in the blue curve. (D) SDS/PAGE analysis of AjFER. Lane 1: protein marker; lane 2: recombinant protein. (E) DLS analysis of AjFER. The red curve is the volume distribution of particles. (F) CD spectrum and the secondary structure of AjFER.

To investigate the evolutionary relationships between AjFER and those of other species, a phylogenetic tree was constructed by the neighbor‐joining method (Fig. 1A). Phylogenetic tree analysis showed that AjFER had a close genetic distance from the marine invertebrate *Dendrorhynchus zhejiangensis* ferritin (DzFer) [34]. Multiple alignment of the ferritin amino acid sequences of *A. japonicus* with other known species indicated considerable consensus sequences among these ferritins (Fig. 1B). The AjFER protein sequence has the following levels of identity with other ferritins: 52.6% (*H. sapiens* L‐chain ferritin: HuLF) [35], 62.8% (HuHF), 60.5% (*Rana catesbeiana* M ferritin: FrogMF) [12], 64.5% (ChF), 60.6% (MjFer), 66.1% (*Sinonovacula constricta* ferritin: ScFer) [36], 67.5% (DzFer), 64.7% [*Phascolosoma esculenta* ferritin (new): Fer147] [37], and 69.9% (*P. esculenta* ferritin: PeFer) [38]. Sequence alignment and prediction of functional domains revealed that AjFER had a highly conserved ferroxidase center with nine ferritins other than HuLF.

The calculated molecular mass of AjFER without a signal peptide was 20.037 kDa, with a respective theoretical pI of 5.41. The fusion protein was purified to homogeneity by Superdex 200 gel filtration chromatography (Fig. 1C). After induced expression, a SUMO‐tagged AjFER subunit with a molecular weight of approximately 35 kDa was obtained, and the molecular weight of the AjFER subunit without a SUMO tag was approximately 20 kDa (Fig. 1D). The results of ICP‐MS indicated that the iron content of purified AjFER was approximately 67 atoms per cage. Subsequently, DLS analysis indicated that only one population centered at 13.54 ± 2.18 nm was observed for the AjFER sample (Fig. 1E). As shown in Fig. 1F, CD analysis revealed that the far‐UV CD spectrum of AjFER exhibited a dominance of the α‐helix structure with broad negative minima at approximately 208 and 225 nm, resulting in a content of 85% α‐helices and 15% β‐turns, excluding β‐sheets and random coils.

### Overall structure of AjFER

**Fig. 2:**
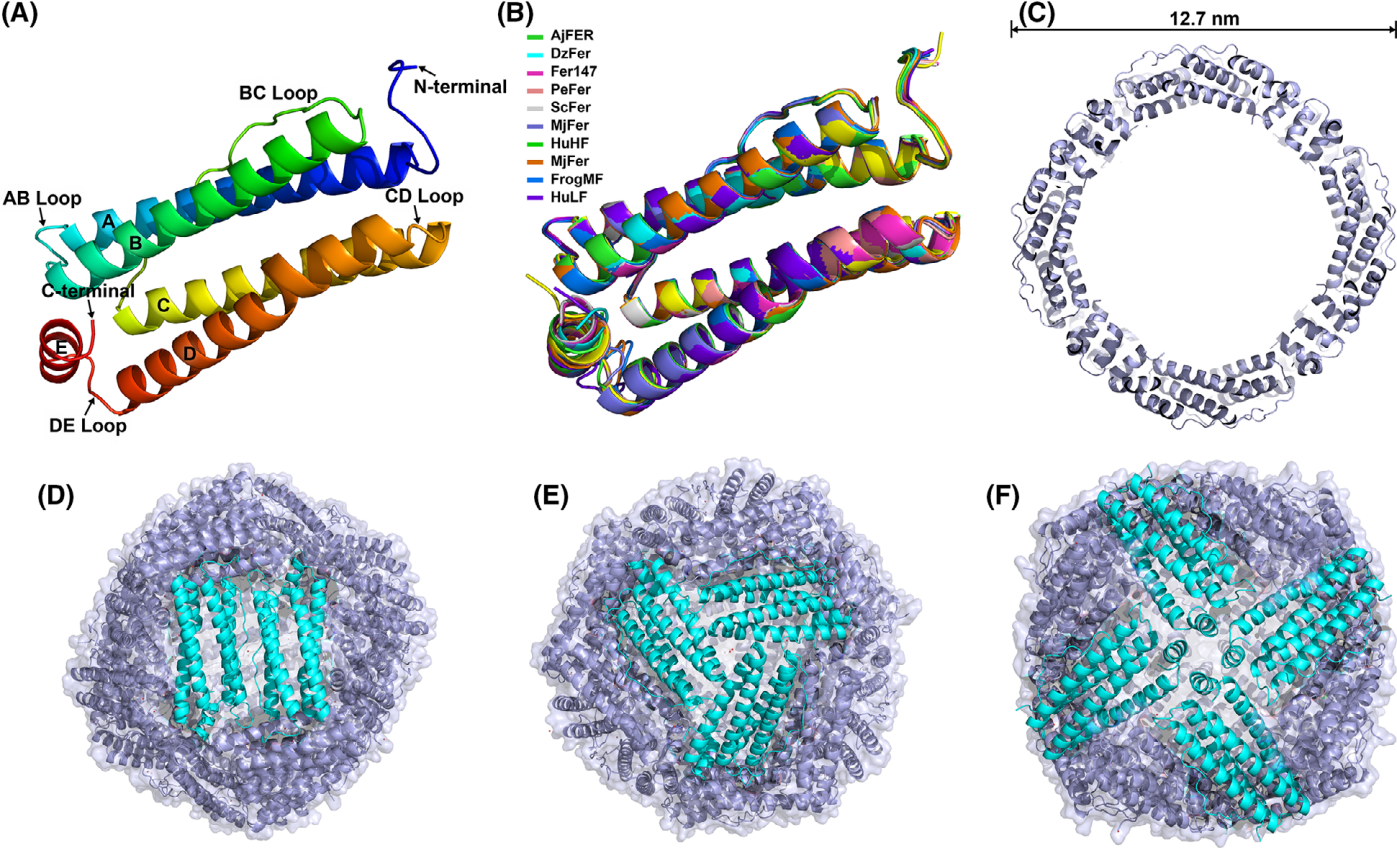
Overall structure of AjFER. (A) The building blocks of the subunit. (B) Structure superimposition for the monomeric ferritin subunit of AjFER and nine ferritins. (C) Cross‐sectional image of the cage‐like cavity of AjFER. Cartoon views of the overall structure viewed from the (D) 2‐fold channel, (E) 3‐fold channel, and (F) 4‐fold channel.

The crystal diffraction data were scaled to a maximum resolution of 2.75 Å with space group P12_1_1, and the crystallographic and refinement statistics are shown in Table 1. In the AjFER structure, each subunit is made up of four long α‐helices (A–D) connected end to end in a parallel antiparallel manner and a short α‐helix (E) inclined to the central axis of the four‐helical bundle at approximately 60° at the C terminus (Fig. 2A). However, due to a lack of electron densities corresponding to the former two amino acid residues (Met1 and Gln2) at the N termini and the last three amino acid residues (Lys171, Glu172, and Asp173) at the C termini, the structure of only 3–170 residues was determined in each subunit of AjFER. The five α‐helices comprise residues 12–38 (helix A), 47–73 (helix B), 94–119 (helix C), 127–154 (helix D), and 160–168 (helix E). Superimposition of these structures indicates that the structure of the AjFER subunit is very similar to that conserved in nine other ferritins (RMSDs of 0.38–0.76 Å) (Fig. 2B). The protein shell of AjFER assembles into a large spherical cage with an outer diameter of 12.7 nm (Fig. 2C). The crystal structure of AjFER presents a 24‐mer protein shell with 2‐, 3‐, and 4‐fold axis symmetry, resulting in twelve 2‐fold channels (Fig. 2D), eight 3‐fold channels (Fig. 2E), and six 4‐fold channels (Fig. 2F).

### Electrostatic potential of AjFER

**Fig. 3:**
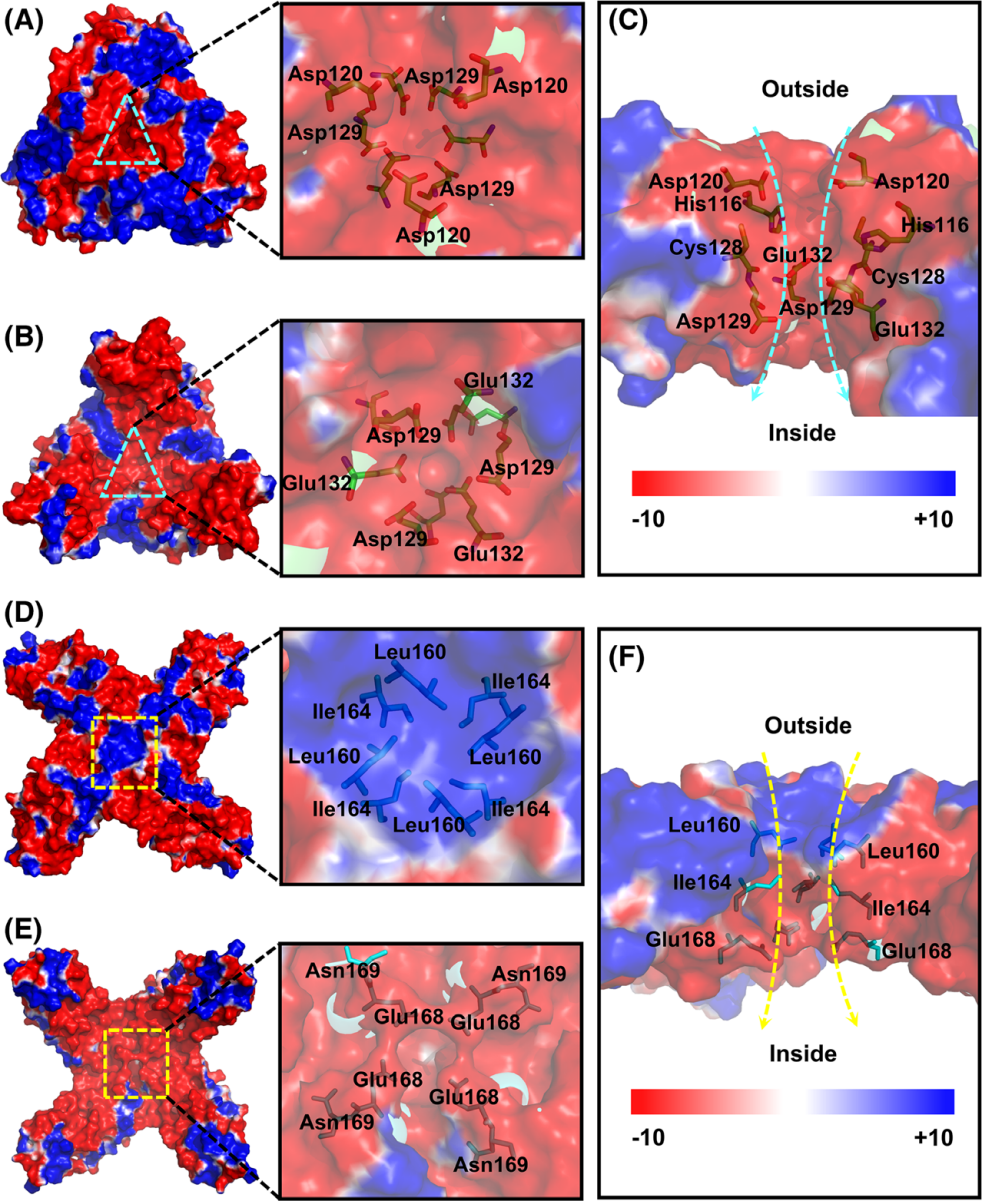
The surface electrostatic potential of AjFER. The electrostatic potential of the 3‐fold channel from the (A) outside, (B) inside, and (C) cross‐section. The electrostatic potential of the 4‐fold channel from the (D) outside, (E) inside, and (F) cross‐section. Surface electrostatic potentials ranging from −10 kT·e^−1^ (red) to +10 kT·e^−1^ (blue) were calculated by APBS.

**Fig. 4:**
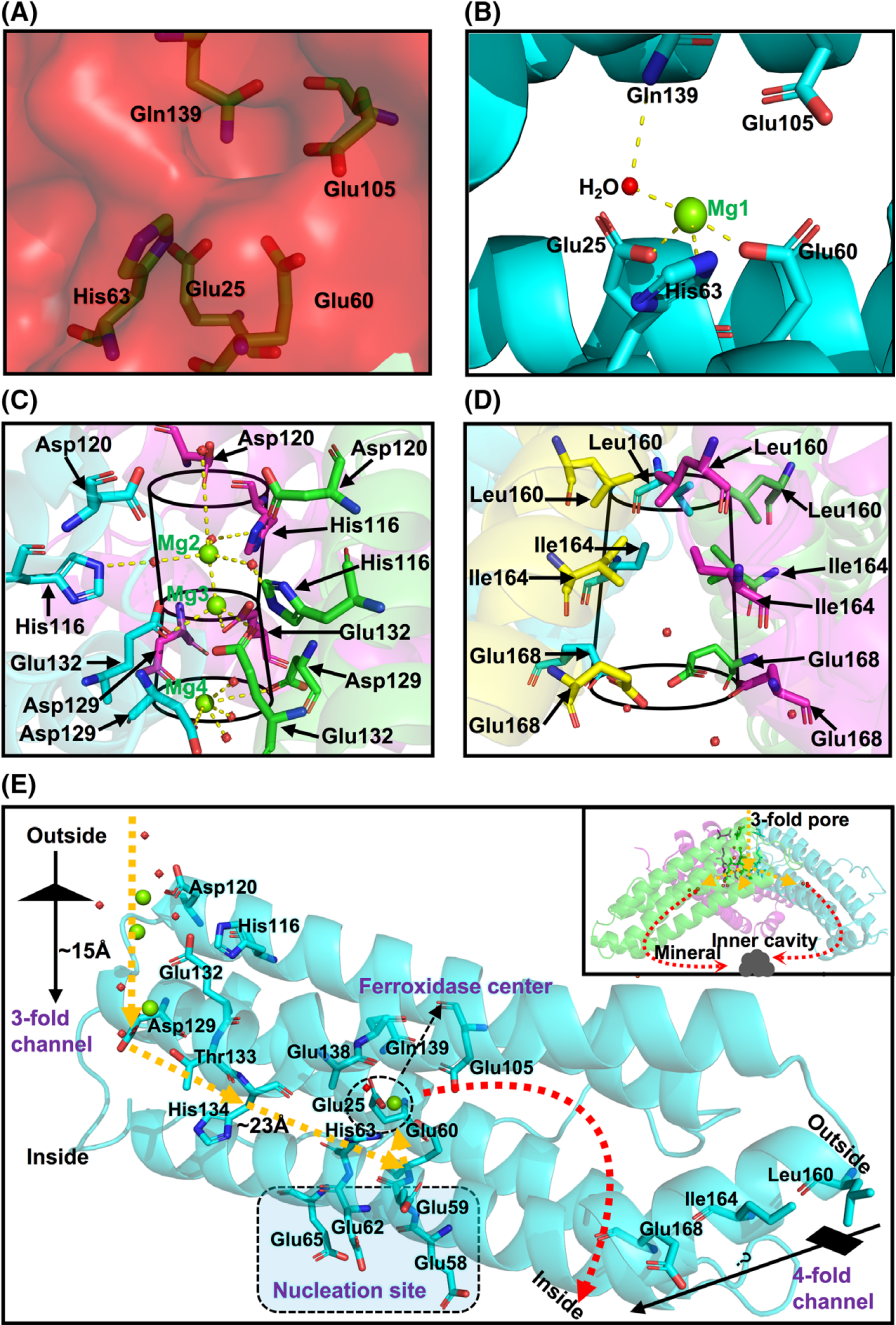
Ion binding sites and transfer path of AjFER. (A) The electrostatic potential of the ferroxidase center. (B) The combination of Mg^2+^ ions is shown in the ferroxidase center. (C) The combination of Mg^2+^ ions was observed in the 3‐fold channel. (D) The key residues of the 4‐fold channel. (E) Transfer path of Mg^2+^ ions from the 3‐fold channel to the ferroxidase center into the cavity. Key residues are highlighted as sticks, Mg^2+^ ions are shown as green spheres, and water molecules are displayed as red spheres.

An examination of the electrostatic potential on both the internal and external surfaces is shown in Fig. 3. The 3‐fold channel is almost surrounded by negatively charged amino acids to form a predominantly negative potential region from outside to inside (Fig. 3A,B). The hydrophilic channels on the 3‐fold channel of AjFER are formed by alternating positively and negatively charged residues (His116, Asp120, Asp129, and Glu132) (Fig. 3C). In contrast to the 3‐fold channel, the regions of the 4‐fold channel outside the entrance show positively charged areas on the pore, yet the inside of the channel entrance exhibits negatively charged residues (Fig. 3D,E). The entrance along the 4‐fold channel forms a hydrophobic line with the side chains of Leu160 and Ile164, and hydrophilic amino acid residue Glu168 is located inside the 4‐fold channel (Fig. 3F). The ferroxidase site of AjFER is predominated by very negative values of the potential, which can mainly be attributed to the residues Glu25, Glu60, and Glu105 (Fig. 4A).

### Metal ion binding sites

The crystals of AjFER were grown in a precipitant solution containing 200 mm magnesium acetate tetrahydrate. Thus, it should seem reasonable that the structure presented in AjFER includes a variable number and type of Mg^2+^ ions from the crystallization buffer via free diffusion [12, 39]. The positions of magnesium ions were assigned based on the strongly positive electron density and the octahedral coordination geometry, that is, the relatively short distance between magnesium ions and ligands (amino acids or water molecules) [31, 40]. At the ferroxidase center, only one Mg^2+^ ion (Mg1) was observed to be trapped (Fig. 4B). Mg1 is ligated by one water molecule at each helix: Glu25 (helix A), Glu60 (helix B), His63 (helix B), and Gln139 (helix D). As shown in Fig. 4C, three Mg^2+^ ions, that is, Mg1, Mg2, and Mg3, were observed in the 3‐fold channel, divided into three layers. The 3‐fold channel in AjFER is 15.0 Å long and positioned around the 3‐fold symmetry axes of the cage. This channel forms a funnel‐like pore with four layers, composed of Asp120, Cys128, Glu132, and Glu129 from the external to internal surface, and the external and internal ion channel pores are 6.7 and 5.1 Å in diameter, respectively. In the entry of the 3‐fold channel, Mg2 is bonded with three water molecules that are coordinated with three residues of His116. Mg3 is coordinated with three adjacent residues of Glu132 in the middle layer, forming Mg–O bonds with a distance of ~ 3.0 Å. On the innermost side of the 3‐fold channel, Mg4 is coordinated with three water molecules and three residues of Asp129, resulting in Mg–O bonds (distance ~ 3.5 Å). The 4‐fold channel in AjFER is 16.7 Å long, forming a trumpet‐like pore that is lined with residues Leu160, Ile164, and Glu168 from the outside to the inside (Fig. 4D). The diameters of the external and internal ion channel pores are 5.4 and 7.7 Å, respectively. The ferroxidase center and 3‐fold channel are composed of negatively charged residues, forming an iron oxidation pathway (Fig. 4E).

## Discussion

In this study, we recombinantly expressed ferritin from the marine invertebrate *A. japonicus* and analyzed the first crystal structure of AjFER. The results showed that the overall structure of AjFER displayed a cage‐like hollow spherical shell composed of 24 subunits. Although the three‐dimensional structural model of AjFER is highly similar to that of other ferritin species, the detailed mechanisms underlying iron storage by AjFER remain relatively unknown.

Evolutionally, *A. japonicus* is affiliated with the phylum Echinodermata, which is one of the two major deuterostomes (the other is the phylum Chordata); thus, Echinodermata is regarded as one of the highest groups in marine invertebrates [19]. Studies on the origin and evolution of the immune system in Echinodermata are always of focus interest in aquiculture [41]. Previous research has shown, like most invertebrates, the possible involvement of ferritin in innate immune defense by an iron‐withholding strategy in *A. japonicus* [19, 42]. Phylogenetic tree analysis revealed that AjFER is genetically closest to DzFer, and the homology analysis of ferritin amino acid sequences suggested that AjFER shares relatively high similarity with these ferritins from invertebrate species (Fig. 1A,B). Furthermore, AjFER exhibited general characteristics, including possessing a relatively conserved structural arrangement of 2‐, 3‐, and 4‐fold channels (Fig. 2D–F). In contrast, the root mean square deviation (RMSD) values of the main chain alpha carbon (Cα) are 0.76 Å between AjFER and HuLF (PDB ID: 2FFX) [35] for 168 residues (52.6% identity over 173 amino acids), 0.61 Å between AjFER and HuHF (PDB ID: 2FHA) [43] for 168 residues (62.8% identity over 172 amino acids), and 0.63 Å between AjFER and FrogMF (PDB ID: 3KA3) [12] for 168 residues (60.5% identity over 172 amino acids). Furthermore, the RMSD values of the main chain Cα are 0.48 Å between AjFER and ChF (PDB ID: 5WPN) [14] for 168 residues (64.5% identity over 169 amino acids) and 0.67 Å between AjFER and MjFer (PDB ID: 6A4U) [15] for 167 residues (60.6% identity over 170 amino acids). The main chain Cα RMSD values between AjFER and ScFer (PDB ID: 6LP5) [36] with 66.1% identity over 171 amino acids are 0.57 Å over 168 residues, while between AjFER and Fer147 (PDB ID: 6LPD) [38] with 64.7% identity over 173 amino acids are 0.55 Å over 167 residues. Of note, the main chain Cα RMSD values between AjFER and DzFer (PDB ID: 7EMK) [34] is 0.73 Å over 167 residues (67.5% identity over 169 amino acids). Moreover, multiple sequence alignment analysis indicated that AjFER shared the highest sequence identity (69.9% over 173 amino acids) with PeFer (PDB ID: 6LPE) [38] compared to the other ferritins (Fig. 1B), and their main Cα RMSD value was 0.57 Å for 168 amino acid residues. These superpositions suggest that AjFER may have a structural and functional characterization similar to that of other ferritins from marine invertebrate species.

Generally, electrostatic gradients are of crucial importance for the overall functioning of proteins [44]. The negative potential on the walls of the 3‐fold channel can provide strong enough attraction for cations to guide them into the protein cavity, whereas the opposite can happen for the 4‐fold channel usually containing predominant positive potential, thereby expelling cations from inside the protein cage [44, 45]. In the structure of AjFER, our calculations showed that the 3‐fold channel and ferroxidase center were conspicuously surrounded by regions that nearly all presented negative electrostatic potential (Figs 3C and 4A). The negatively charged electrostatic potential at the 3‐fold channel across the protein shell to the interior cavity stems from a high density of negatively charged residues, with the main contributors being the highly conserved residue Glu132 and the less conserved residues Asp120 and Asp129 (Fig. 3C), consistent with previous reports [36]. Notably, the carbonyl groups (His120 and Cys128 residues) at the C termini of three subunits can also play crucial roles in forming the predominantly negative potential around the 3‐fold channel. Furthermore, the electrostatic potential at the exterior mouth of the 4‐fold channel is prevalently positive (Leu160 and Ile164 residues), while that of the interior mouth is predominantly negative (Glu168 residue) (Fig. 3F), similar to that previously proposed for HuHF [44]. Moreover, the prevalence of negative electrostatic potential at the ferroxidase center suggested that it exhibited strong iron‐binding capacity, with the major contributors being the highly conserved residues Glu25, Glu60, His63, Glu105, and Gln139 (Fig. 4A). The arrangement of electrostatic potential may contribute to a higher inflow of iron toward the ferroxidase center, suggesting much more efficient ferroxidase activity. Therefore, it can be speculated that the 3‐fold channel likely acts as the major entrance for metal ions to the protein cavity, whereas the 4‐fold channel may be likely to provide some available metal ion binding sites in AjFER.

In the present study, only one large positive peak was proposed to be associated with Mg1 bound in the ferroxidase center (Fig. 4B), and it is coordinated through acidic and alkali groups (Glu25, Glu60, and His63 residues), consistent with a previously reported structure of Mg1 coordinated at the ferroxidase site of FrogMF [12]. It is generally known that the 3‐fold axis develops a very apparent hydrophilic channel that connects the ferritin cage‐like core with the external environment [9]. In the structure of AjFER, the 3‐fold channel shows a funnel shape with a wide entrance at the external part and a relatively narrow pathway (~ 15 Å long) (Fig. 4C), similar to that of frog M ferritin [46]. Interestingly, the channel consisted of three 3‐fold negative amino acid rings from the D helix, including three Asp129 residues forming the inner layer close to the shell interior, three Glu132 residues forming the middle layer, and three Asp120 residues forming the outermost layer before the funnel opening, which is quite distinct from that of other ferritins, apart from ScFer (Figs 1B and 4C). Moreover, it has been reported that for human H‐ferritin (PDB ID: 2FHA), the residues His118 and Cys130 can be able to attract free Fe^2+^ ions, and others, guiding them into the 3‐fold channel [47]. Thus, we suggest that residues His116 and Cys128 of AjFER may play similar roles in the iron acquisition pathway. Additionally, Masuda et al. [48] demonstrated that the residue Glu140, corresponding to the conserved residue Glu138 in AjFER (Fig. 1B), functioned as a crucial transit site of Fe^2+^ ions from the interior exits of the 3‐fold channel to the ferroxidase center. Accordingly, it can be concluded that metal ions in AjFER can be first captured by Asp120 from the outside environment, attracted by His116 and Cys128 when entering the channel, and then transferred by Glu138 from the 3‐fold channel to the ferroxidase site (Fig. 4E). Of note, the 4‐fold channel in AjFER is composed of two hydrophobic rings (Leu160 and Ile164 residues) plus a hydrophilic ring (Glu168 residue) (Fig. 4D), which is largely divergent from the 4‐fold channels composed of two fully hydrophilic (soybean) [49] or fully hydrophobic (*Ulva pertusa*) [50] rings in plant ferritins as well as two fully hydrophilic rings in bacterioferritins [9]. Nevertheless, it is doubtful whether the 4‐fold channel in AjFER can also play a role in counseling a likely route of metal ion entry and release. The putative nucleation site of AjFER is composed of four glutamate residues (Glu58, Glu59, Glu62, and Glu65) (Fig. 4E), which is fully consistent with that of PeFer [38], with corresponding glutamate residues Glu53, Glu56, Glu57, and Glu60 of the HuLF [43] and horse spleen L ferritin (HoLF) [51]. Therefore, the highly conserved residues of AjFER at the nucleation site suggest equivalent capabilities to those of L ferritins.

## Conclusions

In this study, we recombinantly expressed ferritin from the marine invertebrate *A. japonicus* and determined its crystal structure by X‐ray diffraction at 2.75 Å resolution. Our results demonstrated that AjFER showed a 4‐3‐2 symmetry cage‐like hollow shell consisting of 24 subunits, whose structural characteristics were mostly similar to those of other known ferritin species. The 3‐fold channel containing 3‐fold hydrophilic amino acid rings from the D helix was nearly surrounded by regions of very negative potential, suggesting the major entrance of metal ions to the protein cavity. However, the electrostatic properties of the 4‐fold channel suggested a great disadvantage for the passage of metal ions. The crystal structure of AjFER is expected to provide a novel structural model for exploring the mechanism underlying iron storage by marine invertebrates, but further research is necessary to elucidate the detailed mechanisms.

## Conflict of interest

The authors declare no conflict of interest.

## Author contributions

TM and XS conceived and designed the experiments; TM, YW, CH, and XQ collected and analyzed the data; TM and YW performed structure determination and wrote original draft; TM, YW, CS, CL, JZ, YL, and XS made manuscript revisions. All authors contributed to the final manuscript.

## Data Availability

The atomic coordinates and structural factors of AjFER have been deposited in the PDB under the accession code 7VHR.
